# Mechanosensory molecules and circuits in *C. elegans*

**DOI:** 10.1007/s00424-014-1574-3

**Published:** 2014-07-23

**Authors:** William R. Schafer

**Affiliations:** Cell Biology Division, MRC Laboratory of Molecular Biology, Francis Crick Avenue, Cambridge, CB2 0QH UK

**Keywords:** Mechanosensation, *C. elegans*, Nociceptor, Proprioception, TRP channel, Neural circuits

## Abstract

Mechanosensory neurons, whose activity is controlled by mechanical force, underlie the senses of touch, hearing, and proprioception, yet despite their importance, the molecular basis of mechanotransduction is poorly understood. Genetic studies in *Caenorhabditis elegans* have provided a useful approach for identifying potential components of mechanotransduction complexes that might be conserved in more complex organisms. This review describes the mechanosensory systems of *C. elegans*, including the sensory neurons and circuitry involved in body touch, nose touch, and proprioception. In addition, the roles of genes encoding known and potential mechanosensory receptors, including members of the broadly conserved transient receptor potential (TRP) and degerin/epithelial Na^+^ channel (DEG/ENaC) channel families, are discussed.

## Introduction

A number of animal senses, including touch, hearing, and proprioception, involve the control of sensory neuron activity by mechanical force. Because the latency of mechanosensory responses is too rapid to be accounted for by second-messenger signaling pathways, they are inferred to rely on ion channels or channel complexes that are directly gated by force [22]. In contrast to the well-characterized metabotropic sensory transduction pathways involved in vision and olfaction, the mechanisms, and, in many cases, the identities of the molecules mediating ionotropic mechanotransduction are not well understood. In principle, studies in simple genetically tractable model organisms such as *Caenorhabditis elegans* offer an approach to identify molecules involved in ionotropic sensory transduction and study their function in vivo. The existence of a complete connectome [73] for the *C. elegans* neural circuitry also facilitates studies of how mechanosensory information is processed, integrated, and used to control behavior.

Despite the structural simplicity of the *C. elegans* nervous system, at the molecular level the *C. elegans* genome contains representatives of all the known families of channels implicated in mammalian sensory transduction. These gene families show remarkably similar diversity in mammals and nematodes despite the huge differences in anatomical scale [36]. For example, both the human and worm genomes contain six genes encoding cyclic nucleotide-gated channels, which mediate phototransduction and olfactory transduction in mammals and are required for olfaction, thermosensation, and gas sensing in *C. elegans*. Likewise, the worm genome contains 24 transient receptor potential (TRP) channels (comparable to the 24 in the human genome), with representatives of each of the major subfamilies [33, 30]. The degenerin/epithelial Na^+^ channel (DEG/ENaC) channel family, implicated in touch and nociception, is actually more diverse in nematodes than in mammals, with 28 members in *C. elegans* compared to 9 in humans [33]. Thus, *C. elegans* not only is a good model for investigating the properties of known sensory transduction channels, but also may serve as a platform for identifying novel yet undescribed channels with conserved functions in sensory neurons.

This review presents an overview of what is known about *C. elegans* mechanosensation at the molecular and neural circuit levels. The first section surveys the functional roles of the diverse mechanosensory neuron classes and the neuronal circuits within which they act to control behavior. The second part describes what has been learned about the molecular basis of mechanical sensing, in particular the molecules that are known or hypothesized to contribute to ionotropic mechanoreceptor complexes.

## Mechanosensory circuits

Touch and other mechanical senses are critically important for sensory perception in nematodes [31]. Indeed, of the 302 neurons in the nervous system of the *C. elegans* adult hermaphrodite, at least 46 are putative sensory neurons implicated in the detection of attractive or aversive touch stimuli [73]. Of these, around half are involved in sensing body touch and the remainder in sensing touch at or around the animal’s nose. In addition, many neurons, including certain classes of motor neurons and interneurons, are thought to be proprioceptive. The neurons that contribute to these three sensory systems are discussed below.

### Body touch

Nematodes perceive body touch primarily as an aversive stimulus. However, cell ablation, genetic, and physiological studies have shown that gentle and harsh body touch stimuli are sensed differently by *C. elegans* and require different neurons [9, 11, 71]. Of the neurons sensing body touch, the best characterized are the neurons sensing gentle mechanical stimuli. Three of these (the paired ALMs and AVM) evoke a backward escape reflex in response to light touch to the anterior body, and two PLMs) evoke a forward escape reflex in response to posterior body touch [11]. A sixth neuron, PVM, responds to gentle posterior touch [15] but is neither necessary nor sufficient for touch-evoked escape behavior [11, 75]. The receptive fields for anterior and posterior gentle touch neurons are largely nonoverlapping; AVM and the ALMs exhibit neuronal activity in response to touch between the nose and midbody, while PVM and the PLMs respond to touch between the midbody and the tail [65]. Mechanoreceptor currents have been measured directly in one of these neuron classes, the PLMs [57]; these currents are rapidly adapting and show both on and off responses.

A total of 14 neurons have been implicated by cell ablation experiments in the detection of harsh body touch [71, 53]. Best characterized among these are two pairs of multidendritic neurons [58], one with a receptive field covering most of the body (the PVDs) and the other covering the head and neck region (the FLPs) [1]. Both the PVD and FLP neurons respond to fast, high-displacement mechanical stimuli applied to the areas covered by their respective dendritic arbors [17, 16]. The PVDs and FLPs are both polymodal nociceptors, responding to aversive thermal as well as mechanical stimuli [17, 30, 54]. Several additional neurons have been identified that contribute to harsh head touch, including SDQR, AQR, and the paired BDU and ADE neurons [53]. Likewise, the paired PDE, PHA, and PHB neurons contribute to posterior body touch, the latter two classes particularly near the tail itself [53]. In the cases of PVD and PDE, harsh touch-evoked mechanoreceptor currents have been measured by whole-cell patch recording [53].

**Fig. 1:**
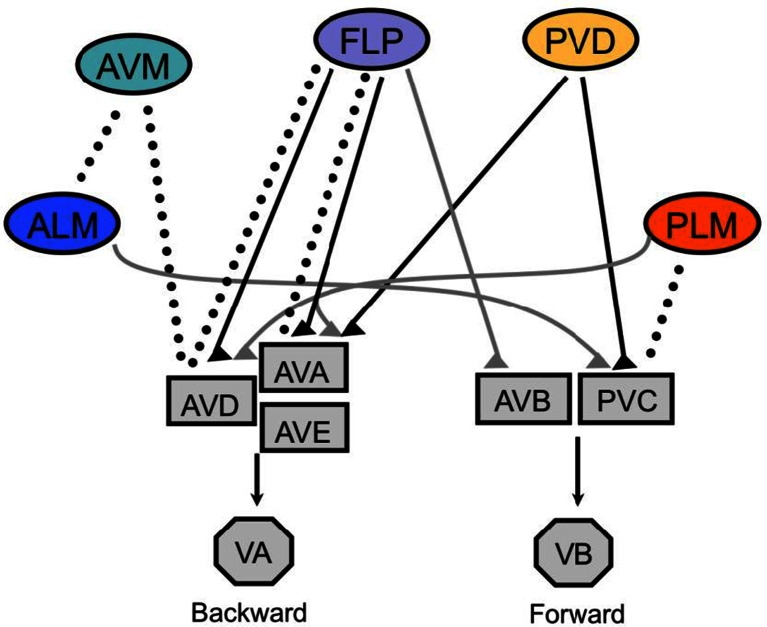
Sensory neurons and interneurons mediating escape responses to body touch. Shown are the connections between sensory neurons (*circles*), interneurons (*rectangles*), and motor neurons (*octagons*) involved in escape responses to body touch. *Dotted lines* indicate gap junctions; *solid lines* indicate chemical synapses (*black lines* are inferred to be excitatory, and *gray lines* are inferred to be inhibitory)

The interneuronal circuitry required to generate escape responses to gentle and harsh touch has also been explored, primarily through cell ablation experiments [11, 74, 76, 53]. Escape behavior in *C. elegans* is linked to a network of five interneuron pairs: AVA, AVD, and AVE, which promote backward locomotion, and AVB and PVC, which promote forward locomotion (Fig. 1). Anterior gentle body touch triggers a switch from forward to backward locomotion; these reversals specifically require the AVD interneurons, which are electrically coupled to AVM (which is itself electrically coupled to the ALMs). Conversely, accelerated forward movement triggered by posterior gentle body touch requires the PVC neurons, which are electrically coupled to the PLMs. Escape responses to harsh touch require same set of neurons, with the addition of DVA which is specifically required for acceleration away from harsh tail touch [53]. Interestingly, this set of neurons corresponds exactly to the *C. elegans* rich club neurons, a network characterized by high degree of connection to other neurons and each other [69]. Thus, body touch information inputs directly into the major center for sensory integration and locomotion control in the worm.

In addition to evoking acute escape responses, body touch also affects other behaviors on a longer time scale. For example, *C. elegans* normally executes head swings, also known as foraging movements, in the course of forward and backward locomotion. Anterior body touch suppresses these foraging movements while the animal is moving backward [2]. Interestingly, other stimuli that evoke similar reversal responses such as nose touch do not suppress foraging, indicating that foraging suppression is a specific response to anterior touch. Touch-evoked foraging suppression requires the tyraminergic RIM interneurons, which inhibit the neck motor neurons and muscles through a tyramine-gated anion channel LGC-55[60]. This behavior may allow nematodes to evade predacious fungi [55].

The body touch receptor neurons, like many *C. elegans* neurons, contain neuropeptides [43, 56, 50]. Though the functional roles of these peptides are mostly unknown, it is likely that these might mediate longer-term effects on behavior. Interestingly, the PVM neuron, which appears to be unimportant for touch-evoked escape behavior, expresses the neuropeptide precursor gene *flp-20* [43], which has recently been shown to be important for control of mechanosensory habituation [51]. In the future, it will be interesting to explore the roles of other touch-neuron-expressed neuropeptides on neuronal plasticity and behavioral states.

### Nose touch

A relatively large number of neurons (at least 26) are involved in sensing mechanical stimuli around the worm’s nose. These include the bilaterally symmetric ASH neurons, polymodal nociceptors that detect aversive chemical, osmotic as well as mechanical stimuli [40]. The FLP nociceptor neurons, in addition to sensing harsh touch to the side of the head as described above, also play an important role in sensing aversive nose touch [40]. Additional nose touch mechanoreceptors are found in labial sense organs with fourfold or sixfold radial symmetry about the worm’s mouth opening. Each of the six inner labial sensilla contains the ciliated ending for one IL1 and one IL2 neuron, while each of the four outer labial sensilla contains the ending for OLQ and a CEP neuron. The CEPs, along with the paired ADE and PDE neurons located in the body, are unusual in that they contain dopamine [64], a key modulator of feeding-related behavioral states [13].

The functions of nose mechanosensory neurons have initially been investigated by cell ablation experiments. When worms collide head-on with an object during forward movement, they usually crawl backward away from the stimulus, an escape behavior similar to that evoked by anterior body touch [21]. Ablation experiments demonstrated that the ASH and FLP neurons are principally required for this response; ablation of either neuron pair alone significantly reduces nose touch-evoked reversals, and ablation of both classes nearly eliminates them [40]. The OLL neurons have also been reported to affect this behavior [12]. Some nose touch stimuli, particularly those to the side of the head, evoke a different avoidance response, designated a head withdrawal, in which the nose itself is retracted from the stimulus through bending of head muscles [35]. Ablation experiments indicate that the OLQ and IL1 neuron classes are required for this response. A third mechanosensory behavior involving nose sensory neurons involves a slowing response when animals sense the texture of a bacterial lawn [62]. Ablation experiments, as well as studies of dopamine-deficient mutants, implicate the dopaminergic CEP and ADE neurons in this response. Finally, *C. elegans* dauer larvae exhibit a behavior called nictation, in which they crawl up fungal hyphae to aid dispersion by insect carriers [49]. Initiation of nictation behavior is triggered by mechanical detection of the hyphal fiber, a process that requires the IL2 neurons. Together, these results suggested that different classes of nose touch mechanoreceptors sense distinct mechanical stimuli and evoke largely distinct behavioral outputs.

**Fig. 2:**
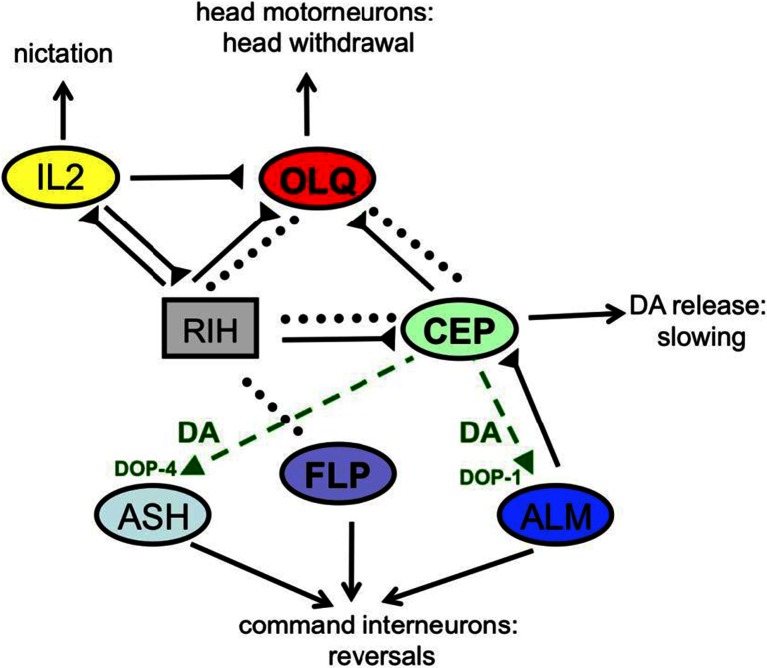
Nose touch sensory circuits. Shown are the connections between a subset of sensory neurons (*circles*) and interneurons (*rectangles*) involved in nose touch. *Dotted lines* indicate gap junctions, *solid lines* indicate chemical synapses, and *dashed green lines* are extrasynaptic connections mediated by dopamine. Behavioral outputs affected by nose touch stimuli are indicated

However, recent experiments reveal a more complex picture, with individual mechanoreceptor classes influencing each other’s activity and impacting multiple behavioral outputs. For example, the FLP, CEP, and OLQ neurons are all electrically coupled through gap junctions to a single hub interneuron, RIH (Fig. 2). In vivo imaging experiments indicate that mechanosensory responses in the OLQ and CEP neurons facilitate gentle touch responses in the FLPs, which normally respond to high-threshold touch stimuli [16]. Conversely, inactive OLQ and CEP neurons act as sinks for neural activity in the nose touch circuit, inhibiting the activity of neurons such as FLP through shunting [61]. Since the FLP neurons make direct synapses with the command neurons (Fig. 2), they serve as an essential link between the RIH-coupled nose touch sensory circuit and the motor circuit that generates escape behavior. In this way, the electrically coupled circuit acts as a coincidence detector, generating a reversal response when all the FLP, CEP, and OLQ sensory neurons are simultaneously active (as in a head-on collision) while triggering alternative behavioral responses (such as head withdrawals) when only some of the mechanoreceptors (e.g., dorsal OLQs) are active.

Interactions between nose touch and other mechanosensory neurons are also mediated by chemical synapses and extrasynaptic modulation. For example, each class of dopaminergic mechanoreceptors receives synaptic input from the gentle body touch receptors with adjacent receptive fields; the CEPs receive excitatory synapses from the ALMs, the ADEs from AVM, and the PDEs from PVM and the PLMs. Conversely, dopamine released from dopaminergic mechanoreceptors acts extrasynaptically on the ALM neurons through the DOP-1 receptor to inhibit sensory adaptation and maintain gentle touch sensitivity [45]. Dopamine likewise acts on the ASH neurons through a different receptor, DOP-4, to sensitize responses to aversive osmotic and chemical stimuli [26]. In this way, the dopaminergic mechanoreceptors promote enhanced sensitivity to aversive stimuli touch and taste exhibited by worms in the presence of a bacterial food source.

### Proprioception

In addition to the neurons that sense external mechanical stimuli, an unknown but significant number of neurons function as proprioceptors that sense body posture. Like other animals, *C. elegans* is thought to use proprioceptive information as sensory feedback to control its pattern of locomotion. In particular, increasing evidence supports a long-standing hypothesis that the processes of several classes of ventral cord motor neurons function as stretch receptors that sense body bending. The B-class motor neurons, which are responsible for forward locomotion [11], contain short anterior-directed processes as well as long posterior-directed processes that extend significantly beyond the region containing synapses with body muscle. These extended processes have been hypothesized to contain stretch receptors involved in sensing body bending [73]. Indeed, a number of theoretical studies have suggested that the sinusoidal body movements underlying nematode locomotion might not require a central pattern generator circuit but instead be generated exclusively by motor neuron sensory feedback [7, 5, 27].

This hypothesis was recently tested experimentally by using microfluidics to enforce specific bending postures and measuring neuromuscular activity with genetically encoded calcium indicators [72]. These experiments demonstrated that body bending induces motor neuron and muscle activity in the segment of the worm immediately posterior to the location of the bend. This implies that the motor neurons must contain stretch receptors that are activated by anterior bending and that this leads to a domino-like propagation of a wave of muscle contraction down the body. Interestingly, both the amplitude and period of the locomotion wave have been shown to be modulated by external forces, for example those related to the viscosity of the medium [4, 27]. These effects might be mediated by additional stretch receptors, possibly in the posterior-directed processes of the motor neurons [5].

Additional examples of proprioceptive neurons in *C. elegans* are the DVA and PVD neurons, both of which have been implicated in the control of body bend amplitude. In the case of DVA, loss-of-function mutations in the mechanosensory TRP channel gene *trp-4* have been shown to act cell autonomously in DVA to cause abnormally deep body bends [52]. In vivo recordings with genetically encoded calcium indicators have shown that DVA is activated by body bending and that this response is dependent on the activity of TRP-4. Similarly, the DEG/ENaC subunit gene *mec-10* has been shown to function in the PVD neurons to alter body bend amplitude [1], and the PVD neurons likewise exhibit bending-activated activity that is *mec-10*-dependent. Neither the DVA nor the PVD neurons are required to generate a locomotion wave, and their ablation has only a modest effect on wave amplitude; thus, the roles of these neurons in proprioception appear to be modulatory. The circuit mechanisms by which these neurons affect body posture are not known.

## Mechanosensory receptors and transduction channels

Perhaps the most compelling reason for studying touch sensation in *C. elegans* is the prospect of using its powerful genetics to identify the long-elusive components of mechanotransduction complexes. Beginning with the classic screens for gentle touch (Mec) mutants [8, 25], forward genetics and phenotyping of knockout strains have identified a number of genes that are required for mechanosensory behaviors. In principle, these genes could encode mechanosensory receptors (Table 1) and/or transduction channels; however, they could also be required more generally for the function of mechanosensory neurons.

**Table 1:** Neurons implicated as mechanosensory receptors

System	Neurons (number)	Implicated behavior
Nose/head	ASH (2)	Nose touch [40]
	OLQ (4)	Nose touch [40], head withdrawal [35]
	OLL (2)	Nose touch [12]
	IL1 (6)	Head withdrawal [35]
	IL2 (6)	Nictation [49]
	CEP (4)	Nose touch [16], food slowing [62]
Body	ALM (2)	Gentle touch [11]
	AVM (1)	Gentle touch [11]
	PLM (2)	Gentle touch [11]
	PVM (1)	Gentle touch [15]
	PVD (2)	Harsh touch [71], proprioception [1]
	FLP (2)	Harsh touch [16, 53], nose touch [40, 16]
	ADE (2)	Harsh touch [53], food slowing [62]
	PDE (2)	Harsh touch [53], food slowing [62]
	SDQR (1)	Harsh touch [53]
	AQR (1)	Harsh touch [53]
	BDU (2)	Harsh touch [53]
	PHA (2)	Harsh touch [53]
	PHB (2)	Harsh touch [53]
Proprioception	DVA (1)	Proprioception [52]
	VB (11)	Proprioception [72]
	DB (7)	Proprioception [72]

To establish a gene product as an authentic sensory receptor, at least three criteria should be met. First, it should be required specifically for neuronal responses to mechanical stimuli. This can be established most straightforwardly by demonstrating a defect in mechanotransduction currents in a loss-of-function mutant. Alternatively, since many *C. elegans* sensory neurons are polymodal, it may be possible to show a defect in touch-evoked activity as measured by optical indicators, with responses of the same neuron to other stimuli unaffected. Second, it is important to demonstrate that the mechanosensitive conductance requiring the putative sensory receptor is biologically relevant. Thus, the gene of interest must be shown to affect a mechanosensory behavior. Finally, an authentic mechanosensor should not only be necessary but also sufficient to respond to mechanical stimuli. This can be demonstrated by reconstituting a functional sensor through expression in a heterologous cell type.

Since mechanosensory responses are very rapid, neuronal mechanoreceptors inferred to be ionotropic. Thus, it is also important to determine whether a candidate receptor gene encodes a pore-forming ion channel. Evidence that a gene encodes a channel can be obtained most straightforwardly by identifying pore mutations that alter the ionic permeability of the mechanotransduction channel in a specific manner. It is worth noting that mechanotransduction may be mediated in some cases by heteromeric protein complexes; thus, the ion channel that mediates the mechanosensory current may not be sufficient to function on its own as a mechanosensor. For example, the transduction channel may be functionally coupled to another molecule that directly senses the mechanical force.

With these criteria in mind, which *C. elegans* genes can be said to encode authentic mechanosensors? Evidence for particular candidates is discussed below.

### Transient receptor potential channels

The *C. elegans* TRP channel family is diverse and contains members of several subfamilies implicated in mechanosensation in other organisms [38]. Of these, the TRPN protein TRP-4 is the one most clearly established as an ionotropic mechanoreceptor. The *trp-4* gene is expressed in several mechanosensory neurons, including the dopaminergic touch receptors (CEP, ADE, and PDE) and the DVA proprioceptive interneurons [52]. *trp-4* is required cell autonomously in the dopaminergic neurons for food slowing and nose touch avoidance behaviors [45, 39] and in DVA for the control of body bend amplitude [52]. In the CEP neurons, *trp-4* is required specifically for calcium responses to gentle but not harsh nose touch stimuli [45]. Electrophysiological recordings from dissected CEP neurons demonstrate that *trp-4* is essential for nose touch-evoked mechanotransduction currents [39]. Crucially, mutations in a putative pore region alter the ionic permeability of the mechanically activated channels in CEP, providing decisive evidence that TRP-4 is a pore-forming ion channel subunit [39]. Although heterologous expression of *C. elegans*TRP-4 has not been reported, its *Drosophila* orthologue NompC has been shown to be sufficient to form mechanically activated channels in other cell types [78]. Thus, based on evolutionary conservation, TRP-4 appears likely to form channels that function as ionotropic mechanoreceptors.

A second TRP channel implicated in mechanosensation in *C. elegans* is TRPA-1. *trpa-1* is required cell autonomously in the OLQ neurons for behavioral responses to nose touch, including head withdrawal and escape [46]. Heterologous expression of *C. elegans* TRPA-1 in mammalian cells leads to the production of pressure-activated ionic currents, suggesting that TRPA-1 is sufficient for the formation of mechanosensitive channels. However, in calcium imaging experiments, *trpa-1* mutations led to only a modest defect in touch-evoked activity in the OLQ [46]. Thus, TRPA-1 may mediate only a minor component of the mechanosensory response in these neurons, with the primary sensory encoded by a different gene. Interestingly, in the multidendritic PVD neurons, TRPA-1 is not required for harsh touch responses at all, but rather functions as a thermosensory receptor for cold [17, 77].

A third TRP channel implicated in mechanosensation is the TRPV channel OSM-9, which is expressed in several touch neurons including ASH, FLP, and OLQ [19]. In ASH, OSM-9 protein is localized to the endings of sensory cilia, and *osm-9* loss-of-function mutants are nose touch insensitive [68]. However, the *osm-9* phenotype in ASH is not specific to touch, as responses to both soluble and volatile chemical stimuli are also impaired in the loss-of-function mutant. Moreover, heterologous expression of *osm-9* expression has not been reported to be sufficient for sensory responses in other *C. elegans* neurons nor in cultured cells. Likewise, nose touch-evoked mechanotransduction currents measured in ASH are unaffected by *osm-9* [29]. Thus, OSM-9 appears unlikely to function directly as a mechanosensor in ASH. OSM-9 may play a more general role in ASH sensory responses, for example as an amplifier of mechanotransduction currents. OSM-9 is also required in the OLQ neurons for calcium and behavioral responses to nose touch [16]; however, at present, no evidence addresses whether it might function as a mechanosensor in these neurons or in a more general capacity. Interestingly, *osm-9* mutations have no cell-autonomous effect on touch responses in the FLP neurons [16], suggesting that it is dispensible even as an amplifier for mechanosensation in these polymodal nociceptors.

### Degerin/epithelial Na^+^ channel

The DEG/ENaC channels comprise a second family of potential ionotropic receptors implicated in mechanosensation in *C. elegans*. The founding members of the family, MEC-4 and MEC-10, were originally identified in forward genetic screens for gentle touch-defective mutants [10]. MEC-4 is specifically expressed in the gentle touch neurons (including PVM), while MEC-10 shows additional expression in the FLP and PVD multidendritic neurons [23, 37]. *mec-4* null alleles are specifically defective in behavioral responses to gentle touch, whereas *mec-4* gain-of-function alleles lead to necrotic neurodegeneration of the touch neurons [8]. The PLM neurons of *mec-4* null mutants completely lack mechanoreceptor potentials, and missense alleles of *mec-4*and *mec-10* have been shown to specifically alter the reversal potential of the mechanotransduction current [57]. These results argue strongly that MEC-4 and MEC-10 are components of channel complex mediating mechanoreceptor currents in touch neurons.

However, while MEC-4 and MEC-10 are clearly components of the gentle touch mechanotransduction channel, the mechanisms by which these channels are gated by mechanical stimuli remain unclear. To date, no reconstitution experiments demonstrating sufficiency of MEC-4 containing complexes for mechanical sensing have been reported. Heterologous expression of mutant forms of MEC-4 (encoded by the alleles causing neurodegeneration) in *Xenopus* oocytes leads to the production of sodium channels, whose activity is enhanced by coexpression of other mec genes, including MEC-10, MEC-2 (a stomatin homologue), and MEC-6 [32, 18]. However, neither these complexes, nor complexes containing wild-type MEC-4 protein, have been reported to be mechanically activated. Moreover, neither *mec-4* nor any other DEG/ENaC family member has been shown to confer mechanosensory responses when expressed in a heterologous cell type. Thus, additional proteins may be required to detect the mechanical stimuli that gate MEC-4-containing channels. These may include proposed extracellular tethers such as MEC-1 or MEC-9 [24] or alternatively may involve other, as yet unidentified sensor proteins.

MEC-10 is also implicated in mechanosensation in the FLP and PVD multidendritic neurons. In the FLPs, MEC-10 is required for behavioral responses to nose touch and harsh head touch, as well as for calcium responses to these stimuli [16]. Since *mec-10* does not affect FLP responses to thermal stimuli, its function appears to be specific to mechanosensation. *mec-10* mutations also affect PVD calcium responses to harsh body touch but not to temperature, and although they do not cause a harsh touch behavioral phentoype on their own [3], *mec-10* and *mec-4(d)* double mutants (which lack the gentle touch neurons) show decreased harsh touch sensitivity [17]. *mec-10* also affects the proprioceptive function of PVD, as *mec-10* mutants show abnormal locomotion waveforms and lack bending-evoked calcium transients [1]. MEC-10 has not been reported to form channels when expressed heterologously in oocytes [32, 18], suggesting that it may require association with a second DEG/ENaC protein such as MEC-4 to generate a functional channel. RNAi experiments suggest that another DEG/ENaC protein, DEGT-1, may function together with MEC-10 in both FLP and PVD [17]. However, although these results suggest a specific role for MEC-10/DEGT-1in harsh touch mechanotransduction, *mec-10* mutants show robust mechanotransduction currents in PVD [53]. Thus, MEC-10-containing channels may play an indirect role in touch sensing in these neurons as has been suggested for DEG/ENaC channels in the mammalian dorsal root ganglion (DRG) [22].

DEG/ENaC channels have also been implicated in nose touch detection in the ASH neurons. Mutations in the *deg-1* gene dramatically reduce the magnitude of ASH mechanotransduction currents evoked by nose touch [29]. Moreover, mutations analogous to those previously shown to alter the reversal potential of MEC-4/MEC-10 channels had similar effects on the reversal potentials observed for the ASH mechanotransduction currents. These results demonstrate that DEG-1 is a pore-forming subunit of mechanically sensitive channels in ASH. Interestingly, the abnormalities in nose touch avoidance behavior seen in *deg-1* mutants or in animals treated with the potent DEG/ENaC blocker amiloride are relatively modest (approximately 20 % reduced) compared to the defects reported to result from cell-specific ASH ablation (around 60 % reduced) in other studies [29, 40]. Since the *deg-1* mutant alleles tested in these behavioral assays were not null, it is possible that residual DEG-1 activity in mutant and amiloride-treated animals accounts for at least some of this apparent difference. Alternatively, an additional, DEG/ENaC-independent mechanosensory modality in ASH may make a biologically relevant contribution to nose touch behavioral responses.

Two additional DEG/ENaC channels, DELM-1 and DELM-2, have been implicated in nose touch sensation. Loss-of-function mutations in either gene result in a nose touch behavioral defect and are reduced response to repeated nose touch stimulation in the OLQ neurons [34], a phenotype similar to that of the *trpa-1* deletion mutant [46]. However, *delm-1* and *delm-2* are expressed not in the OLQ neurons, but in associated glial cells, and the nose touch phenotype of the *delm-1* and *delm-2* mutants can be rescued by glial-specific expression of the cogate wild-type transgene. Moreover, the requirement for *delm-1* and *delm-2* can be bypassed by glial-specific ectopic expression of an inwardly rectifying potassium channel, expected to increase K^+^ extrusion from the glial cell into the fluid surrounding the OLQ cilium [34]. Thus, DELM-1 and DELM-2 do not appear to function as direct mechanical sensors in the OLQs, but rather act in glial support cells, most likely to enhance OLQ excitability. A closely related DEG/ENaC gene, *acd-1*, plays a similar role in amphid glial cells, facilitating chemosensory responses in the ASH neurons [70].

The *C. elegans* genome contains many more DEG/ENaC family members (28 in total), but the functions of most of these are still unknown. One body muscle-expressed DEG/ENaC protein, UNC-105, has been suggested as a possible contributor to stretch receptors [28]. However, although *unc-105* gain-of-function alleles lead to muscle hypercontraction, no locomotion phenotype has been described for the *unc-105* null allele. The product of another DEG/ENaC gene, *unc-8*, has been proposed as a possible proprioceptor based on its expression in motor neurons and its effect on the locomotion waveform. However, the locomotion phenotype of *unc-8* null mutants is relatively subtle [66], suggesting that the stretch receptor that propagates the locomotion wave is probably encoded by a different gene. High-throughput phenotyping of strains carrying deletions in a number of other DEG/ENaC channel genes identified abnormal locomotion waveforms consistent with a role in proprioception [79]; however, since these analyses were conducted on single alleles, these phenotypes must be considered preliminary. Interestingly, deletion mutants in two DEG/ENaC genes, *asic-2* and *acd-5*, show highly specific and nearly identical abnormalities in turning behavior [6]; it is interesting to speculate that these genes may encode subunits of a channel involved in proprioception in the nose.

### Other candidate receptors and future perspectives

In *C. elegans*, as in mammals and flies, the TRP and DEG/ENaC channel families have been implicated as key components of mechanotransduction complexes. However, there are also multiple examples of mechanosensory neurons in which the mechanism of sensory transduction is unknown; in particular, the stretch receptors involved in proprioception remain unidentified as do the molecules in neurons such as ASH that sense osmotic stimuli. It is not unreasonable to suppose that at least some of these processes might be mediated by additional classes of functionally conserved mechanoreceptors or mechanotransduction complexes. The *C. elegans* genome contains homologues of most of the ion channels found in mammals, some of which might be good candidates for mechanotransducers. For example, *C. elegans* contains an orthologue of Piezo, a mechanotransduction channel involved in touch sensing in mammals and flies [20, 44], though no mechanosensory role for this gene has been reported.

Another family of channel-like proteins recently implicated as potential mechanotransduction channels is the transmembrane channel-like (TMC) family. TMC genes encode multipass integral membrane proteins and are broadly conserved in animals [48, 42]. Vertebrate genomes contain eight TMC genes, while *C. elegans* contain two and *Drosophila* one. The human *Tmc1* is a major deafness gene [47], and mouse *Tmc1* is required together with its paralogue *Tmc2* for cochlear hair cell mechanotransduction [41, 59]. However, neither gene has been shown to be sufficient to generate mechanical responses or channel activity in a heterologous system [41]. In *C. elegans*, one the TMC genes, *tmc-1*, is specifically required for sodium chemosensation in the ASH neurons and is sufficient to function as a sodium sensor in worm olfactory neurons and cultured mammalian cells [14]. Although channel activity has not been conclusively demonstrated, these results are consistent with TMC-1 functioning as an ionotropic receptor for salt. Expression profiling studies have shown that the other *C. elegans* TMC gene, *tmc-2*, is expressed in mechanosensory neurons [63]. Thus, TMC-2 is a plausible candidate for a mechanoreceptor channel in these cells.

Despite vast differences in nervous system scale, there appears to be significant conservation at the molecular level between the sensory transduction mechanisms of worms, flies, and mammals; indeed, most of the known families of transduction channels (TRPs, cyclic nucleotide-gated channels, DEG/ENaCs, Piezos, TMCs) are well-conserved. In principle, it may be possible in the future to identify additional families of conserved sensory receptors and transduction channels using *C. elegans* genetic screens. With the recent availability of collections of sequenced, heavily mutagenized strains [67], reverse genetic screens may provide an effective approach, especially in polymodal neurons such as ASH where it essential to identify mutations with touch or osmotic-specific defects. Machine vision approaches for high-content behavioral phenotyping [79] may also make it possible to discern precise alterations in locomotion pattern expected for mutants with abnormalities in proprioceptors or neuromuscular stretch receptors. By identifying *C. elegans* receptors for these and other poorly understood mechanosensory modalities, we may ultimately hope to identify cognate receptors for other organisms, including humans.
